# Dual-NMS: A Method for Autonomously Removing False Detection Boxes from Aerial Image Object Detection Results

**DOI:** 10.3390/s19214691

**Published:** 2019-10-28

**Authors:** Zhiyuan Lin, Qingxiao Wu, Shuangfei Fu, Sikui Wang, Zhongyu Zhang, Yanzi Kong

**Affiliations:** 1 Shenyang Institute of Automation, Chinese Academy of Sciences, Shenyang 110016, China; 2 Institutes for Robotics and Intelligent Manufacturing, Chinese Academy of Sciences, Shenyang 110169, China; 3 University of Chinese Academy of Sciences, Beijing 100049, China; 4 Key Laboratory of Opto-Electronic Information Processing, Chinese Academy of Sciences, Shenyang 110016, China; 5 The Key Lab of Image Understanding and Computer Vision, Shenyang 110016, China

**Keywords:** false detection boxes, density of detection boxes, dual-NMS, object detection, aerial image, deep learning

## Abstract

In the field of aerial image object detection based on deep learning, it’s difficult to extract features because the images are obtained from a top-down perspective. Therefore, there are numerous false detection boxes. The existing post-processing methods mainly remove overlapped detection boxes, but it’s hard to eliminate false detection boxes. The proposed dual non-maximum suppression (dual-NMS) combines the density of detection boxes that are generated for each detected object with the corresponding classification confidence to autonomously remove the false detection boxes. With the dual-NMS as a post-processing method, the precision is greatly improved under the premise of keeping recall unchanged. In vehicle detection in aerial imagery (VEDAI) and dataset for object detection in aerial images (DOTA) datasets, the removal rate of false detection boxes is over 50%. Additionally, according to the characteristics of aerial images, the correlation calculation layer for feature channel separation and the dilated convolution guidance structure are proposed to enhance the feature extraction ability of the network, and these structures constitute the correlation network (CorrNet). Compared with you only look once (YOLOv3), the mean average precision (mAP) of the CorrNet for DOTA increased by 9.78%. Commingled with dual-NMS, the detection effect in aerial images is significantly improved.

## 1. Introduction

Object detection is a fundamental task in the area of computer vision. Its purpose is to identify the categories of objects in an image and obtain the locations of each object. In recent years, with the rapid development of deep learning, various types of high-performance image segmentation and object detection networks have been proposed. The commonality of the abovementioned networks is that they all employ the powerful feature extraction capabilities of convolutional neural networks. The regions with convolutional neural network (R-CNN) was a pioneering work on applying neural networks in the field of object detection, R-CNN’s phased design idea essentially reflected its utilization of the convolutional neural network’s powerful feature extraction ability [1]. The network structure can be simplified by integrating different parts of the R-CNN [2,3]. The reason for simplifying the network structure is that the convolutional neural network can autonomously extract features, and also has strong fitting ability. Neural networks can map highly abstract features to object categories, locations and even depth information [4,5]. The one-stage object detection networks that were proposed in [6,7] validate the above viewpoint. The anchor-free network structure that was proposed in [8,9,10] makes full use of the neural network’s fitting ability.

**Figure 1:**
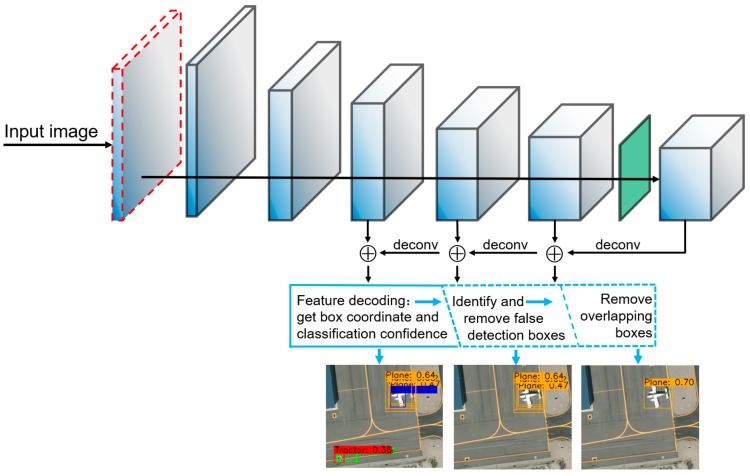
The detection network structure. The upper side is the CorrNet for extracting the features of the input images. The lower side is the post-processing part of the detection results. The blue dotted box is the dual-NMS.

Although the convolutional neural network already has strong feature extraction capabilities, it is difficult to achieve a further improvement in its detection precision, especially in specific scenes such as aerial images. Because objects are relatively small in aerial images, their features cannot be fully extracted, which makes it difficult for the classical object detection network to effectively distinguish the objects to be detected from the background. In addition, a large number of detection boxes are incorrect. For the above problems, two methods can be used to improve the detection precision of the object detection network in specific scenarios. (1) The network’s ability to extract object features can be enhanced by optimizing the network structure. It fundamentally improves the performance of the object detection network in specific scenarios. This correspond to the feature extraction process on the upper side of Figure 1. (2) The decoding process from the feature maps to the detection results and the post-processing method could be optimized. This corresponds to the post-processing process in the blue box at the bottom of Figure 1. The existing algorithms mainly optimize these two stages in the object detection process. The ability of the network to extract object features is enhanced by fusing different levels of feature maps [11,12,13,14]. By deepening and widening the network, more abstract features can be obtained to improve the classification accuracy [15,16,17]. References [18,19,20] mainly improve the accuracy by optimizing the decoding process of the network. References [21,22,23,24] improved the NMS to adapt to more complex detection scenarios. In the small object detection of aerial images, the features of the objects that are to be identified are difficult to extract, and objects and backgrounds are indistinguishable; therefore, there are many false detection boxes in the detection results, which directly affect the precision of the detection network. The existing methods do not optimize the detection network from the perspective of removing false detection boxes. If the false detection boxes can be accurately deleted, the detection precision will naturally be improved.

To solve the problems that were mentioned above, the dual-NMS is proposed in this paper, which corresponds to the blue dotted box in Figure 1. Considering the density of the detection boxes for each detected object and the classification confidence of the corresponding detection boxes, the false detection boxes are adaptively removed, which greatly improves the detection precision without changing the detection network structure. In addition, the detection network is optimized to fundamentally enhance its ability to extract object features. The detection network: you only look once (YOLOv3) accomplishes object classification and location simultaneously in the process of convolution [25]. Unlike two-stage detection network, YOLOv3 completes object detection in forward convolution process, so it has faster detection speed. Compared with other one-stage object detection networks, YOLOv3 has a better detection effect. Therefore, the research work of this paper is based on YOLOv3. The dilated convolution guidance structure is added to the initial part of YOLOv3, which allows the network to better extract the overall features of the objects. The loss of small objects in the feature maps is avoided by reducing the downsampling times of the detection network. References [26,27,28] calculate the correlation of the image contents, which improves the segmentation accuracy of the network. However, they use the entire feature tensor to calculate the correlation. These methods recombine the separated foreground and background features and reduce the ability of the network to distinguish individual objects. In this paper, the correlation calculation layer is introduced in the detection network, and the feature channels are separated from each other. By calculating the correlations between similar objects, the correlation calculation layer allows the network to more easily to detect small objects in aerial images. These optimization methods that were mentioned above make up the CorrNet. The process of extracting the features of the input image by using the CorrNet and removing the false and overlapped detection boxes by using the dual-NMS are shown in Figure 1. The structure that is shown in Figure 1 has achieved better results for the VEDAI, RSOD [29], UCAS-AOD and DOTA [30] aerial image object detection datasets. Our main contributions can be summarized as follows:

1) The dual-NMS is proposed. As an optimization algorithm for detection results, the precision of the object detection results is greatly improved under the premise of keeping the recall unchanged.
2) We proposed the CorrNet. The feature extraction capability of the convolutional network is enhanced by introducing the correlation calculation layer of the feature channel separation and the dilated convolution guidance structure. This enhancement makes the CorrNet more suitable for the detection of small objects in aerial images.
3) Experiments were carried out by using several aerial image object detection datasets to verify the effects of the dual-NMS and CorrNet, and the results were compared with those of YOLOv3. The experimental results show that the CorrNet has better detection effects for the small objects in aerial images.

The remainder of this paper is organized as follows: Section 2 briefly reviews the related works, and Section 3 details the dual-NMS and the optimized detection network: CorrNet. The experimental results and methods are given in Section 4, which is followed by the conclusions in Section 5.

## 2. Related Works

In the existing object detection networks, after the convolution and feature extraction of the input images, the categories and positions of each object in the images are obtained by decoding the feature maps, and a series of detection boxes are generated in the meantime. In the Faster-RCNN [3], YOLOv3 [25], SSD [7] and some other detection networks, the detection boxes with higher classification confidence by default have higher positioning accuracies. This hypothesis is discussed in [18], the classification confidence and positioning accuracy of the detection boxes are not strictly linear, and the positioning score of the detection boxes is taken as the optimization condition. When the detected objects are dense, removing false detection boxes by using the NMS will lead to missed detections. By reducing the classification confidence of the highly overlapped detection boxes, missed detections can be avoided to a certain extent [21]. Reference [22] used the Kullback–Leibler divergence loss to measure the distribution loss between the prediction boxes and the ground truth, which improves the localization accuracy of the detection boxes. Reference [23] increased the flexibility of the NMS by training a network to set different intersection-over-union (IoU) thresholds for detection boxes with different IoUs. The NMS is realized through the trained network, which makes it more suitable for dense scenes with high occlusion [24]. The NMS is optimized for text detection in natural scenes [31,32,33]. The abovementioned post-processing methods optimized the detection results by removing the overlapped detection boxes. Similarly, the detection results can be optimized by removing false detection boxes.

In addition to optimizing the post-processing of detection results, improving the feature extraction capability of the convolutional network is the fundamental solution to improving object detection. There are many optimization methods for the convolution process. Under the same number of parameters and computational complexity, better overall features can be obtained by using the dilated convolution [34,35]. References [36,37,38,39] apply different weights to the feature map through an attention mechanism so that the convolution networks could autonomously focus on the key objects in images. In aerial image object detection, because the objects are small, it is difficult to extract the features during object detection. A continuous convolution and pooling process will lead to the disappearance of small objects in high-level feature maps. However, the dilated convolution can be used to extract features in low-level convolution layers to obtain the overall features of a single object. To avoid the loss of small objects in the feature map due to excessive downsampling, the downsampling times need to be adjusted. The correlations of the same contents in an image can be calculated, which improves the segmentation accuracy of the network [26,27,28]. Similarly, the correlation at different positions in a single feature map can be calculated, and similar objects can be integrated through the correlation. When combining with the dual-NMS, the false detection boxes are removed, and the detection efficiency of small objects in aerial images is improved by the object detection network.

## 3. Our Methods

The main idea of our method is to autonomously remove false detection boxes from the detection results according to the density of the detected boxes and optimize the object detection network according to the characteristics of aerial images. The corresponding methods are the dual-NMS and the CorrNet respectively. In this section, the proposed dual-NMS algorithm and the CorrNet are analyzed in detail.

### 3.1. Dual-NMS

#### 3.1.1. The False Detection Boxes

**Figure 2:**
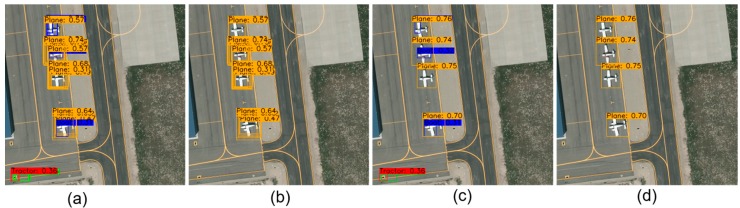
Comparison of the results of the classical NMS and dual-NMS. (**a**) Outputs of the detection network with no post-processing operations. (**b**) Detection results where the false detection boxes have been removed by the dual-NMS. (**c**) The classical NMS algorithm removes the overlapped detection boxes, but fails to remove the false detection boxes. (**d**) The detection results that were processed by the dual-NMS, where the false detection boxes have been correctly removed.

In aerial images, the images are obtained from the top-down perspective, there are fewer features that can be used for object recognition, and the directions of the objects are random; therefore, false detection boxes are more likely to appear. The false detection boxes mainly include two types as shown in Figure 2a: (1) the background is misidentified as an object, such as the Tractor (red box); and (2) the real objects are misclassified, such as the Boat (blue box).

#### 3.1.2. The Differences between True and False Detection Boxes

To autonomously remove the false detection boxes, the density of the detection boxes is defined as the condition that judges whether an object is falsely detected. The density of the detection boxes can be explained in detail as follows. As shown in Figure 2a, there are dense boxes around each aircraft (yellow boxes). In other words, if the object is correctly detected, more detection boxes will be generated around an object. If the detected object is a false detection, the detection boxes that are generated around the detected object are sparse, and the classification confidence of the detection boxes will be smaller, as shown in Figure 2a with the Tractor (red box) and Boat (blue box). These sparse boxes are likely to be false detection unless they have high classification confidences. Therefore, the density of the detection boxes is defined as the number of detection boxes around each detected object. The average densities of the detection boxes that are generated around the true and false detected objects in the output results of the detection network are shown in Table 1. By comparison, the average density of true detection boxes is approximately 3 times that of false detection boxes. Therefore, the density of the detection boxes can be used as a condition to suppress the false detection boxes before the NMS is executed.

#### 3.1.3. Removing the False Detection Boxes by Using the Dual-NMS

The false detection boxes are difficult to effectively eliminate by using post-processing algorithms such as the NMS, and the method still leaves the highest classification confidence false detection boxes, such as the Tractor (red box) and the Boat (blue box) in Figure 2c. If these false detection boxes can be autonomously removed, the precision of the detection results will be greatly improved.

The proposed dual-NMS takes the density of the detection boxes as the condition to remove some detection boxes. Especially in aerial image object detection, it can adaptively identify and remove the false detection boxes. The dual-NMS is divided into two parts: (1) judging whether the current boxes are detected by errors according to the density of the detection boxes and, if so, deleting them; and (2) removing the highly overlapped detection boxes. The results of the dual-NMS adaptively identifying and removing the false detection boxes is shown in Figure 2b, where the false detection boxes have been removed, such as the Tractor (red box) and the Boat (blue box). The dual-NMS is shown in Algorithm 1.


**Algorithm 1: Dual-NMS**
**Input**: ***B*** = {*b*_1_, …, *b*_N_}, ***S*** = {*s*_1_, …, *s*_N_}, *N*_t1_, *N*_t2_, *α*, *β*, *γ* ***B*** is a set of predicted bounding boxes for class A ***S*** is a set of corresponding classification confidences *N*_t1_ and *N*_t2_ are the NMS thresholds *α*, *β* and *γ* are the parameters in function *f* that are used to suppress false predicted bounding boxes **Output**: ***D*** the final predicted bounding boxes 1: ***D*** = ∅ 2: **while**
*B* ≠ ∅ **do** 3:     *b*_t_ ← the first element in ***B***; ***T*** = ∅; ***S*_p_** = ∅ 4:     ***T*** ← ***T*** ∪ {*b*_t_} 5:     ***S*_p_** ← ***S*_p_** ∪ {*s*_t_} 6:     ***B*** ← ***B*** \ {*b*_t_} 7:     ***S*** ← ***S*** \ {*s*_t_} 8:     **for**
*b*_i_ ∈ ***B* do** 9:      **if** IoU (*b*_t_, *b*_i_) ≥ *N*_t1_
**then** 10:       ***T*** ← ***T*** ∪ {*b*_i_} 11:       ***S*_p_** ← ***S*_p_** ∪ {*s*_i_} 12:       ***B*** ← ***B*** \ {*b*_i_} 13:       ***S*** ← ***S*** \ {*s*_i_} 14:      **end if** 15:     **end for** 16:     *S*_sum_ ← *sum* (***S*_p_**) 17:     *d* ← *len*(***T***) 18:     *s_m* ← *argmax* (***S*_p_**) 19:     **if**
*S*_sum_ > *f* (*d*, *s_m*, *α*, *β*, *γ*) **then** 20:      **while *T*** ≠ ∅ **do** 21:       *s*_m_ ← *argmax* (***S*_p_**) 22:       ***T*** ← ***T*** \ {*t*_m_} 23:       ***S*_p_** ← ***S*_p_ \** {*s*_m_} 24:       ***D*** ← ***D*** ∪ {*t*_m_} 25:       **for**
*t*_i_ ∈ ***T* do** 26:        **if** IoU (*t*_m_, *t*_i_) ≥ *N*_t2_
**then** 27:         ***T*** ← ***T*** \ {*t*_i_} 28:         ***S*_p_** ← ***S*_p_ \** {*S*_i_} 29:        **end if** 30:       **end for** 31:      **end while** 32:     **end if** 33: **end while** 34: **return**
***D***

In Algorithm 1, the classical NMS corresponds to lines 20~34, which remove the highly overlapped detection boxes. The dual-NMS corresponds to lines 2~19, which determine whether the detection boxes are false detections according to the density of the detection boxes. The implementation can be divided into three steps:

1) All the detection boxes are grouped according to the IoU between them (lines 3~15). The detection boxes with a certain degree of overlap are divided into a group.
2) The density of the detection boxes in each group is counted. The sum and the maximum of the classification confidences in each group of the detection boxes are calculated.
3) The dynamic threshold that is calculated by function f and the above statistics are used to determine whether the detection boxes in this group are false or not. If they are false detection boxes, delete them.

**Figure 3:**
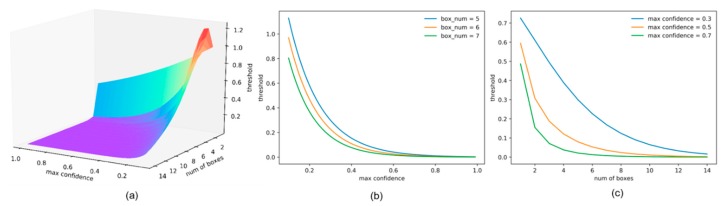
(**a**) Dynamic threshold distribution of the classification confidence and density of the detection boxes. (**b**,**c**) are the univariate relationships of the thresholds for the maximum classification confidence and the density of the detection boxes, respectively.

The dynamic threshold calculation function f has the following form:

(1) $$f\;\left(d,\;s\_m,\;\alpha ,\;\beta ,\gamma \right)=\beta \times e^{-s\_m}\times d^{1-\alpha \times s\_m-\gamma \times d},$$

where d is the density of the detection boxes of the current group, s_m is the maximum value of the classification confidence in the current group, and α, β and γ are the corresponding scaling factors. If the density of the detection boxes is larger, the possibility of real objects appearing at this position is greatly increased. When there are detection boxes with high classification confidence in the current group, even if the density of the detection boxes is lower, the possibility of detecting real objects is also greatly increased. In these cases, dynamic thresholds should be rapidly lowered to avoid blindly deleting true detection boxes. The low density of the detection boxes indicates that the possibility of a false detection is larger, and the dynamic threshold should be kept at a larger value to autonomously remove the false detection boxes. Based on the above analysis,$\;e^{-s\_m}$ ensures that the threshold decreases as the maximum classification confidence increases. $d^{1-\alpha \times s\_m-\gamma \times d}$ guarantees that the threshold decreases as the density of the detection boxes increases, and it also controls the action intervals of different thresholds. The distributions of f with respect to the density of the detection boxes and the maximum value of the classification confidence are shown in Figure 3. The dynamic threshold decreases as the density of the detection boxes and the maximum classification confidence increase. The sum of the classification confidence of all detection boxes reflects the density of the detection boxes and the reliability of a correct detection. By comparing the dynamic threshold with the sum of the classification confidences of all the detection boxes, the process of judging whether the current group of boxes is a false detection is realized according to the density of the detection boxes. After removing highly overlapped detection boxes using the classical NMS, the whole dual-NMS process is completed. The above procedure can adaptively identify and remove the false detections and avoid incorrect removals of true detection boxes. The corresponding experimental validation is described in detail in Section 4.2. The values of α, β and  γ are experimentally determined in Section 4.3. The classical NMS algorithm can be replaced with the soft-NMS in the subsequent part of the dual-NMS to make the algorithm more adaptable.

### 3.2. CorrNet

#### 3.2.1. The Characteristics of Aerial Images

Most of the existing object detection networks are trained on datasets such as VOC and COCO. The structure of the detection network should match the characteristics of the datasets. Compared with VOC and other datasets, the scale of the objects in aerial images is much smaller. In the VEDAI, RSOD, UCAS-AOD and DOTA datasets, the proportion of objects with different scales to all targets is shown in Table 2. The scales can be divided into four levels: *S*_1_ ≤ Min (*H*, *W*)/32, *S*_2_ ≤ Min (*H*, *W*)/16, *S*_3_ ≤ Min (*H*, *W*)/8 and *S*_4_ ≤ Min (*H*, *W*)/4. *H* and *W* are the length and width of the input image, respectively. The scale (S) is the larger value of the object’s length or width. In the datasets, more than 90% of the objects have scales below 1/8 of the input image’s length and width. In addition, in VEDAI, 67.85% of the object scales are less than 1/32 of the input image’s length and width. The statistical results show that small objects account for the vast majority in the four datasets. It makes detection network difficult for the existing object detection models to achieve the ideal detection results in aerial image datasets.

In the object detection network, the input image is continuously convoluted and subsampled to get the feature map of the input image. Then, the feature maps are decoded to get the categories and the box coordinates of the objects in the image [1,2,3,6,7]. The max-pooling operation reduces the resolution of the image and increases the receptive field of the convolution kernel. Continuous max-pooling results in a rapid decrease of the image resolution, which makes small-scale objects rapidly disappear in feature maps, and leads to the lack of small object information in highly abstract feature maps. For the above reasons, the key to improve the detection network’s ability to detect small objects in aerial images is to avoid losing small objects in the convolution and pooling processes, and to extract the overall and highly abstract features of the small objects at the bottom of the detection network.

#### 3.2.2. The Optimization Methods

**Figure 4:**
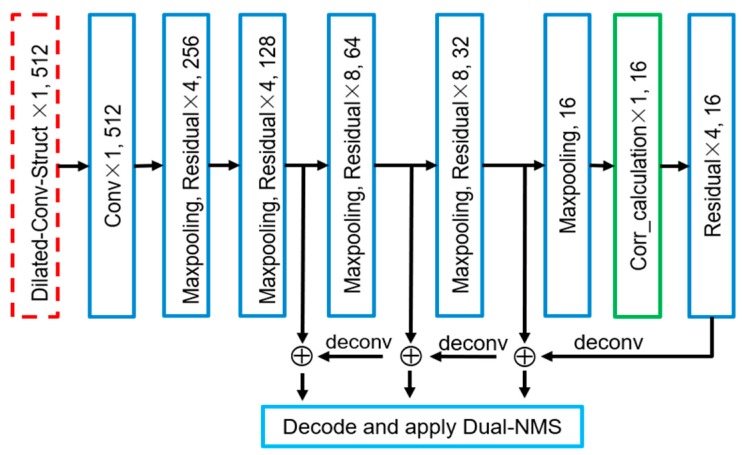
CorrNet structure. The red dashed box indicates the dilated convolution guidance structure. The green box represents the correlation calculation layer of the feature channel separation. The downsampling rates of the feature map of the original YOLOv3 for decoding the calculation loss is 8, 16 and 32, which makes it easy to cause small object losses.

By improving the resolution of the feature maps that are used to calculate the losses, the problem of small objects disappearing in these feature maps can be solved. The resolutions of the feature maps that are used to calculate the losses in the CorrNet are twice those of YOLOv3. Therefore, the downsampling times in the CorrNet are half those of YOLOv3. The overall structure of the detection network is shown in Figure 4. In the CorrNet, feature maps with lower resolutions are upsampled and fused with high-resolution feature maps to compensate for the lack of high abstract features. Through the above improvements, the disappearance of small objects in high-level feature maps is avoided. However, the receptive field of the convolution kernel in the high-level convolutional layers is reduced, and the detection network’s ability to extract the overall features of objects is weakened, which indirectly affects the detection effect of the network.

**Figure 5:**
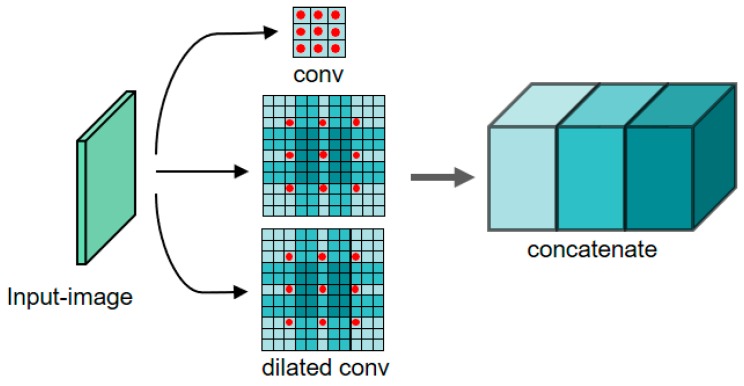
Dilated convolution guidance structure.

To enhance the ability of the convolution network to extract the overall features of the object in the image, in the CorrNet, the input image first passes through the dilated convolution guidance layer, which is the red dashed box in Figure 4. The implementation is shown in Figure 5. The input image is subjected to the conventional convolution and two parallel dilated convolutions, and the results of the three convolution operations are concatenated for the subsequent feature extraction. The receptive field of the convolution kernel is increased by the dilated convolution guidance structure to enhance the network’s ability to extract the overall features of the objects. These two methods greatly improve the detection network’s ability to extract image features, thereby making the detection network more suitable for small object detection in aerial images. The experimental effects and parameters are detailed in Section 4.4.

**Figure 6:**
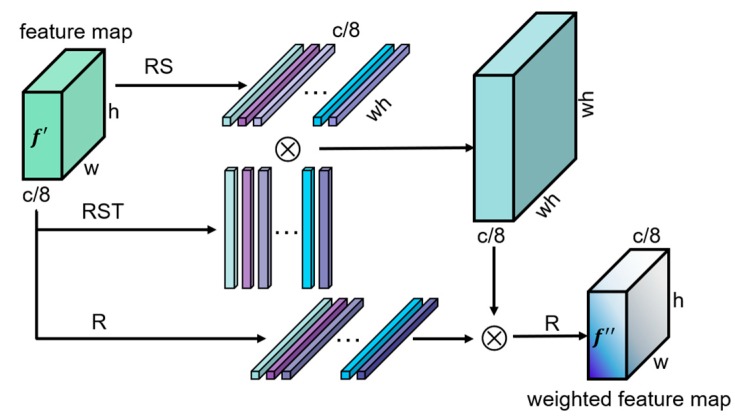
Correlation calculation layer in which the feature channels are separated.

In aerial images, only the top of the object can be seen; therefore, the foregrounds and the backgrounds are indistinguishable. In this case, the fine-grained features of the object and the correlation between objects of the same category are the key to detecting objects. Hence, the correlation calculation layer in which the feature channels are separated from each other is added to the CorrNet. The correlation calculation layer is represented by the green box in Figure 4, and its implementation is shown in Figure 6. The different channels of the feature map contain the features of different positions in the image [36], so the internal relevance of each feature map is calculated in the correlation calculation layer. To reduce the computational complexity, the correlation calculation layer is added to the feature maps with the lowest resolution. For the feature map f with ***C*** input channels, first, the number of channels is reduced to 1/8, and the feature map $f^{\prime }$ is obtained. Then, the *R*, *S* and *T* operations are performed on $f^{\prime }$ in turn. *R*, *S* and *T* correspond to the order modification, mean normalization, and transposition operations of the matrix, respectively:

(2) $$RS\left(f^{\prime }\right)=\;R{\left(f^{\prime }-\bar{f^{\prime }}\right)}_{w\times h}={\left(f^{\prime }-\bar{f^{\prime }}\right)}_{1\times wh},$$

(3) $$RST\left(f^{\prime }\right)=\;RS{\left(f^{\prime }\right)}^{T}={\left(f^{\prime }-\bar{f^{\prime }}\right)}_{1\times wh}{}^{T},$$

(4) $$R\left(f^{\prime }\right)=\;R{\left(f^{\prime }\right)}_{w\times h}={f^{\prime }}_{1\times wh},$$

After the above transformation, the weight of each feature map is obtained by the following formula:

(5) $$W_{wh\times wh}=softmax{\left(RST\left(f^{\prime }\right)\times RS\left(f^{\prime }\right)\right)}_{wh\times wh}\;,$$

Then, the corresponding weight is applied to each feature map to obtain the weighted feature map $f^{\prime \prime }$:

(6) $$f^{\prime \prime }=R{\left(R\left(f^{\prime }\right)\times W_{wh\times wh}\right)}_{w\times h}\;,$$

Finally, the number of $f^{\prime \prime }$ channels is expanded to ***C*** and connected to the original feature map f as the final feature output. The above process calculates the correlation of similar image contents in a single feature map, and it generates corresponding weights for each channel, which improves the ability of the network to detect small objects in aerial images. Section 4.4 will give the experimental results of network structure optimization methods and compare the detection performance of the CorrNet with other detection networks.

## 4. Experiments and Results

To verify the effectiveness of the dual-NMS and the network optimization methods, experiments were carried out on four aerial image object detection datasets, including VEDAI, UCAS-AOD, RSOD and DOTA. The detection results of the original YOLOv3 were used as the baseline, and several evaluation metrics were analyzed. All the experiments were accomplished on a computer with a GTX 1080Ti GPU and a 4.0 GHz CPU. All programs are performed on Ubuntu 14.04 via Tensorflow1.0 and Python3.5. The initial learning rate is set to 0.0001. The optimizer of the network is Adaptive Moment Estimation (Adam), which can automatically adjust the learning rate for each parameter to be learned. Because of the limitation of memory, the batch size is set to 2. When the network achieves the optimal detection effect, the number of epochs is 70.

In the four aerial image datasets, the images in VEDAI are the clearest, with a total of 9 categories to be detected. There are 1087 images as training set and 123 images as test set. On UCAS-AOD and RSOD, the number of images in the training set is 1354 and 841, and the number of images in the test set is 156 and 95. The two datasets contain two and four categories of objects to be detected, respectively. The DOTA dataset contains the largest number of object categories, totaling 15 categories. The size of the image in the DOTA dataset is different, and the high-resolution images make the network difficult to train. We cropped the image to 1024 × 1024 and remove the images containing incomplete targets. Finally, in the DOTA dataset, the training set contains 2287 pictures and the test set contains 254 pictures. In the training process of the detection network, the resolution of all the input images was resized to 512 × 512. Different datasets have different training time. The training time in DOTA datasets is 9 h.

### 4.1. The Metrics that Were Used in the Experiments

Appropriate metrics can reflect the pros and cons of the algorithm. In this paper, the precision, recall, *F*_1_ score, removal rate (Rr), frames per second (FPS) and the mean Average Precision (mAP) are selected as the metrics to evaluate the effectiveness of our methods. The mAP is the average precision at different recalls (0.0~1.0) in all categories. The FPS is the number of frames that the program can process per second. It is an important standard to measure the efficiency of the algorithm.

The precision and recall are defined as follows:

(7) $$Precision=\;\frac{TP}{TP\;+\;FP},$$

(8) $$Recall\;=\;\frac{TP}{TP\;+\;FN},$$

where TP: the number of true objects that are correctly detected in the detection results, FP: the number of objects that are incorrectly identified as objects and falsely classified in the detection results, and FN: the number of objects that failed to be correctly detected.

The *F*_1_ score combines the precision and recall, and it is defined as follows:

(9) $$F_{1}=\left(1+\beta^{2}\right)\cdot \frac{precision\;\cdot \;recall}{\beta^{2}\;\cdot \;precision\;+\;recall\;},\;\beta =\;1,$$

Except for the precision, the recall and *F*_1_ score are used to evaluate the dual-NMS, and the effect of the dual-NMS on the true and false detection boxes was measured by the Rr of the detection boxes in the experiment. The removal rate is defined as follows:

(10) $$Rr=\;\frac{1}{C}\sum_{i=1}^{C}\frac{N_{i}-R_{i}}{N_{i}},$$

where C is the number of object categories in the dataset, and $N_{i}$ and $R_{i}$ are the numbers of detection boxes of class i in the results that are obtained by the classical NMS and the dual-NMS, respectively.

### 4.2. The Effectiveness of the Dual-NMS

**Figure 7:**
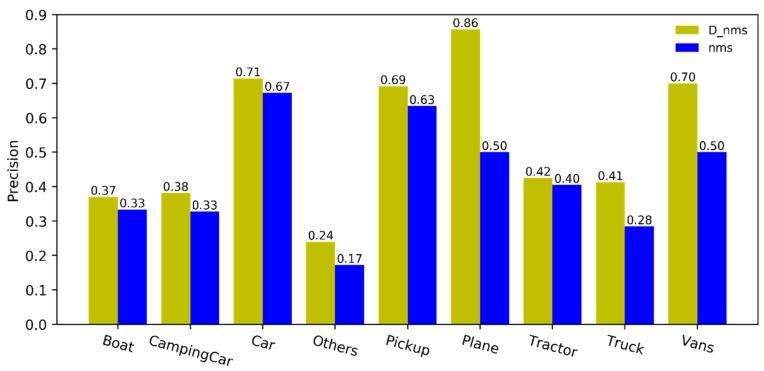
Precision comparison of the detection results after processing by the dual-NMS and NMS, respectively.

**Figure 8:**
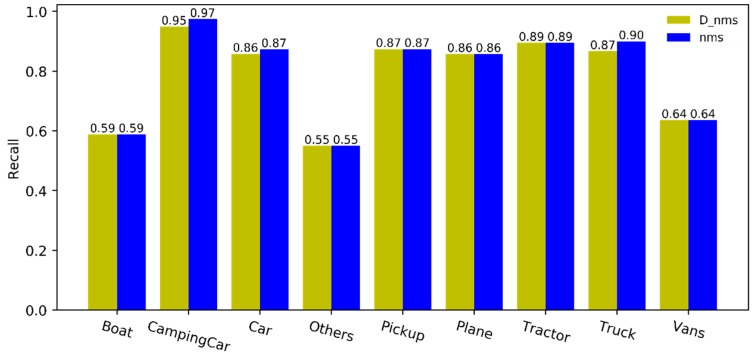
Recall comparison of the detection results after processing by the dual-NMS and NMS, respectively.

As the post-processing algorithm of detection results, the dual-NMS can adaptively remove the false detection boxes, while avoiding any impact on true detection. The precision, recall, and *F*_1_ score take into account the number of true and false detection boxes in the detection results. Because the dual-NMS can autonomously remove the false detection boxes, consequently, the FP is reduced. In addition, attention should be paid to the impact on the correct detection objects, that is to say, the recall should remain unchanged. The precision and recall of the dual-NMS and classical NMS in the VEDAI are shown in Figure 7 and Figure 8, respectively. It can be seen that the precision of the detection results is greatly improved by the dual-NMS. Only the recall of CampingCar, Car, and Truck are slightly lower.

The *F*_1_ score can more objectively and comprehensively reflect the quality of the detection results. The *F*_1_ score of the dual-NMS and classic NMS in different datasets are compared as shown in Table 3. The detection results that are optimized by the dual-NMS are significantly better. The experimental results show that the dual-NMS can adaptively remove the false detection boxes and minimize the impact on the correct results, which proves the effectiveness of the dual-NMS.

The Rr synthetically reflects the ability of the dual-NMS to remove the detection boxes. The max/min removal rates of the true and false detection boxes in each dataset of YOLOv3 and the CorrNet are shown in Table 4. In the two detection networks, although there are errors in removing the true detection boxes, the effect of removing false detection boxes is more significant. The removal rate of false detection boxes (*Rr_F_*) in each dataset is obviously higher than that of true detection boxes (*Rr_T_*). It is still possible to remove more than 10% of the false detection boxes, with a minimal impact on the correct detections. Therefore, the dual-NMS can be used as a general-purpose post-processing algorithm in existing detection networks.

### 4.3. Ablation Experiment

**Figure 9:**
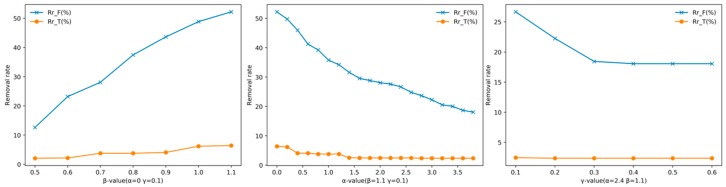
Trend of the removal rate with different *β*, *α* and *γ* values.

**Figure 10:**
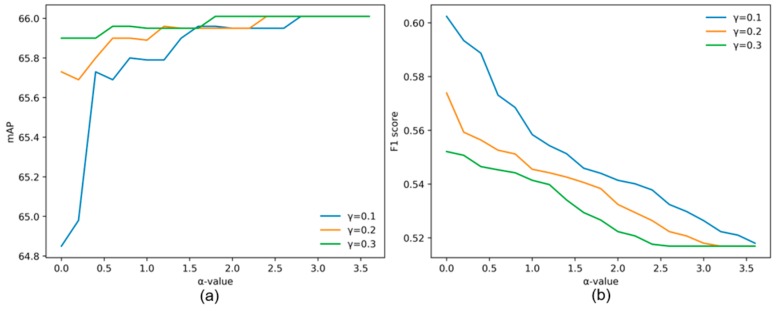
(**a**) Trend of the mAP with different *α* and *γ* values. (**b**) Trend of the *F*_1_ score with different *α* and *γ* values.

In the dual-NMS, different control parameters affect the dynamic threshold. The control parameters indirectly determine the ability of the dual-NMS to suppress false detection boxes and impact true detections. A good control factor should allow the dual-NMS to remove the false detection boxes to the greatest extent, while avoiding impacting true detections. In this section, VEDAI is selected as the test dataset. To reduce the amount of experimental calculations, the correlation calculation layer is not added to the model. The changing trend of the true and false detection box removal rates with *β*, *α* and *γ* are shown in Figure 9. The removal rate increases as *β* increases and decreases as α and *γ* increase. The overall trend of the removal rates accords with the distribution form of the dynamic threshold. The changing trends of the mAP and *F*_1_ score under different *α* and *γ* values are shown in Figure 10. The removal rate of the false detection boxes is the most obvious when *γ* is less than 0.3; therefore, the value of *γ* in Figure 10 is set from 0.1 to 0.3. The overall value of the dynamic threshold is affected by *β*. According to the removal rate of false detection boxes, *β* is directly set as 1.1. In Figure 10, the mAP increases *α* increases, while the *F*_1_ score shows a downward trend. It indicates that the removal rate of the false detection boxes is larger when α is small, and the dual-NMS has a better inhibitory effect on false detection boxes, but excessive suppression of the true detections leads to a decreased mAP. Finally, when the control coefficients are selected as *α* = 2.6 and *γ* = 0.1, the negative effect of the dual-NMS on true detections is the smallest and the false detection boxes are eliminated to some extent.

### 4.4. Analysis of the Effectiveness of the Network Optimization Methods

The role of each optimization method in different aerial image object detection datasets is shown in Table 5. The experimental results are compared with the results of YOLOv3. The resolutions of the feature maps that used to calculate the loss in YOLOv3 are 16 × 16, 32 × 32 and 64 × 64. Conversely the resolutions are set as 32 × 32, 64 × 64 and 128 × 128 in the CorrNet, and the downsample rates are changed from 8, 16, and 32 to 4, 8, and 16. If the down-sample rate is set 32, the objects with a scale less than 32 pixels will disappear in the feature map that with the lowest resolution. It can be seen from the *S*_2_ in Table 2, if the resolution of the input image is 512 × 512, the length and the width of most objects are less than 32 pixels. In this case, it is difficult to effectively detect the objects on the lowest resolution feature map. Therefore, the downsample rate in the CorrNet is changed to half that of YOLOv3. By this method, the disappearing of small objects in the feature maps is avoided effectively, and the computational complexity does not increase significantly. The effect of reducing downsample rate is most obvious in the DOTA datasets, the mAP is increased by 7.4%.

The addition of the dilated convolution guidance structure solves the problem that the receptive field of convolution kernel decreases due to the reduction of downsample rate, and enhances the ability of the convolutional network to extract the overall features of objects. When the dilated rate is set 3, the detection network has the best detection effect. In the VEDAI and DOTA datasets, the mAP improves by 4.09% and 8.8%, respectively. The experimental results show that the overall features play important roles in identifying different objects in images.

The correlation calculation layer of the feature channel separation uses the correlation between the objects of the same category as the weights of the corresponding feature layers. Through the correlation between the same category of objects, the same category of objects is fused into one, which reduces the difficulty of small object detection in aerial images. In particular, the VEDAI and DOTA datasets have more object categories to be detected, and the mAP is improved by 5.19% and 9.78%, respectively. The correlation calculation layer in the UCAS-AOD dataset results in an inconspicuous improvement. The reason is that there are only two categories in the dataset, and there is only a single object category in an image, therefore, the effect of the correlation calculation layer of the feature channel separation is limited.

In Table 6, the detection performance of the CorrNet and other detection networks in four aerial image datasets is compared. The CorrNet has better detection effect in VEDAI, UCAS-AOD and RSOD. In the dataset of DOTA, the performance of two-stage detection network is obviously better than that of one-stage. Especially the Faster-RCNN, the mAP of its detection results exceeds more than 10% of the one-stage detection network. The reason for this phenomenon is that there are more categories in the DOTA dataset, and the classification of targets in the two-stage detection network is accomplished by independent branches. But in other datasets, the CorrNet achieves better detection results with more concise network structure.

The dual-NMS is applied in the YOLOv3 and the CorrNet, respectively, and the mAP and FPS of the detection results in each dataset are shown in Table 7. Compared with the NMS, the application of the dual-NMS makes the mAP slightly lower. This is because the dual-NMS will erroneously remove a very small number of true detection boxes when removing false detection boxes. According to the mAP calculation method, the reduction of the recall will have a serious impact on the mAP. Therefore, the max/min removal rates of the dual-NMS for false and true detection boxes are calculated in Table 4. The effect of the dual-NMS on false detection boxes is much greater than that on true detection boxes. This result has great practical significance in applications requiring strict precision. Further research on how to reduce the impact of the dual-NMS on true detections will be the focus of subsequent work. In this paper, the computational efficiency of the dual-NMS and the CorrNet is evaluated by FPS. From Table 7, it can be seen that the dual-NMS has little effect on the computing time of the detection network, which is less than 0.5 FPS. CorrNet’s FPS is slightly lower than YOLOv3, and the loss of computational efficiency is due to the increase in computational complexity caused by the correlation layer. Although FPS decreased slightly, the mAP of the CorrNet increased significantly.

### 4.5. Comparison of the Detection Results

**Figure 11:**
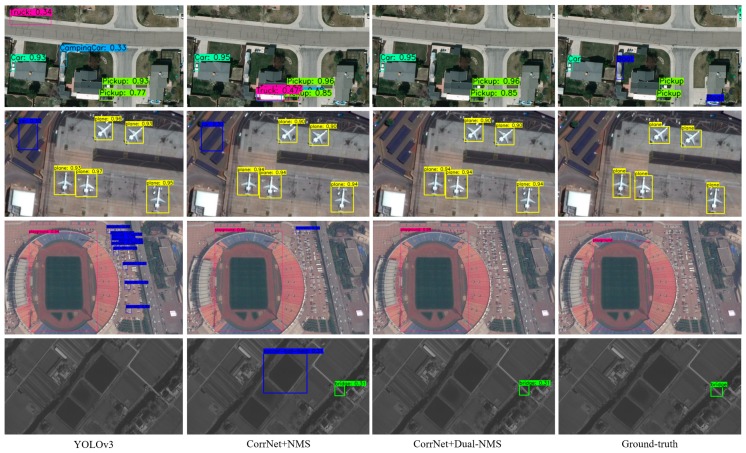
Comparison of the detection results of different network structures. From top to bottom, the results are for the VEDAI, UCAS-AOD, RSOD and DOTA datasets.

In the previous experimental part, the effectiveness of the dual-NMS and the optimization methods of the network structure are analyzed through specific experimental data. The results of the dual-NMS and different network structure optimization methods are intuitively presented in Figure 11. The comparison of the 1st and 2nd columns in Figure 11 shows that the detection result of the optimized detection network is obviously superior to that of the original detection network. For example, the pink box (Truck) at the top of the 1st row and the blue box (aircraft) in the 3th row are significantly smaller than the results of YOLOv3. In the 4th row, YOLOv3 did not detect any objects. Comparing the 2nd and 3rd columns, the false detection boxes have been removed by the dual-NMS to some extent, such as the pink box (Truck) in the 1st row, the blue box (car) in the 2nd row, the blue box (aircraft) in the 3rd row, and the blue box (soccer-ball-field) in the 4th row. The comparison results fully indicate that the CorrNet can better detect small objects in aerial images, and the dual-NMS can autonomously identify and remove the false detection boxes.

## 5. Conclusions

In this paper, the proposed dual-NMS considers the density of the detection boxes and the corresponding classification confidence. The dual-NMS can adaptively identify and remove the false detection boxes. The precision of object detection results is greatly improved under the premise of keeping the recall unchanged. For the VEDAI and DOTA datasets, the removal rate of false detection boxes is more than 50%. The proposed CorrNet combines the dilated convolution guidance structure with the feature channel separation correlation calculation layer, which fundamentally improves the ability of the network to detect small objects in aerial images. In the CorrNet, the mAP is increased by 9.78% for the DOTA dataset. In further research, the recognition results of false detection boxes can be used as feedback information for network training so that the detection network can automatically identify and remove the false detection boxes in the convolution process.

## Figures and Tables

**Table 1:** The average density of the true and false detection boxes in different datasets.

Dataset	Density (True Detection Boxes)	Density (False Detection Boxes)
VEDAI	13.43	5.58
UCAS-AOD	20.08	6.29
RSOD	21.61	7.86
DOTA	12.18	3.85

**Table 2:** The proportion of objects with different scales to all targets in each dataset.

Dataset	*S*_1_	*S*_2_	*S*_3_	*S*_4_
VEDAI	67.85%	97.56%	99.76%	99.97%
UCAS-AOD	15.78%	54.67%	90.87%	96.60%
RSOD	16.01%	77.73%	99.32%	100.00%
DOTA	14.31%	70.07%	100.00%	100.00%

**Table 3:** Comparison of the *F*_1_ score for the dual-NMS in different data sets (The best result in each dataset is marked in bold).

Model	VEDAI	UCAS-AOD	RSOD	DOTA
NMS	0.5392	0.9200	0.7861	0.3356
Dual-NMS	**0.5842**	**0.9495**	**0.7966**	**0.3842**

**Table 4:** Max/min removal rates of the true and false detection boxes for each dataset in different detection networks.

Model	*Rr*	VEDAI	UCAS-AOD	RSOD	DOTA
YOLOv3	*Rr_T_*	11.54%/1.81%	2.88%/0.86%	2.81%/0.18%	25.53%/8.50%
	*Rr_F_*	56.92%/15.42%	57.94%/30.05%	51.02%/8.89%	52.89%/32.19%
CorrNet	*Rr_T_*	6.42%/2.09%	1.01%/0.15%	0.79%/0.08%	11.06%/2.66%
	*Rr_F_*	52.22%/16.22%	40.44%/28.05%	23.47%/14.37%	61.56%/22.10%

**Table 5:** Effectiveness of different optimization methods in each dataset (The best results are marked in bold).

Model	Reduced Downsampling Rates	Dilated Convolution Guidance Layer	Correlation Calculation Layer	mAP (%)			
				VEDAI	UCAS-AOD	RSOD	DOTA
**YOLOv3**				62.86	95.99	83.23	38.91
CorrNet	√			65.57	96.47	85.86	46.31
	√	√		66.95	**97.14**	86.21	47.71
	√	√	√	**68.05**	96.82	**88.02**	**48.69**

**Table 6:** Comparative detection performance of the CorrNet and other detection network (The best results are marked in bold).

Model	mAP (%)			
	VEDAI	UCAS-AOD	RSOD	DOTA
Faster-RCNN	64.70	89.00	84.47	**60.46**
R-FCN	61.80	93.50	84.07	52.58
SSD	46.10	78.88	87.85	29.86
YOLOv2	50.30	89.41	87.35	39.20
YOLOv3	62.86	95.99	83.23	38.91
CorrNet	**68.05**	**96.82**	**88.02**	48.69

**Table 7:** The effects of the dual-NMS on the mAP and FPS in different detection networks.

Model	NMS	Dual-NMS	mAP (%)				FPS
			VEDAI	UCAS-AOD	RSOD	DOTA	
YOLOv3	√		62.86	95.99	83.23	38.91	11.25
		√	62.19	95.32	82.80	37.54	11.04
CorrNet	√		68.05	96.82	88.02	48.69	9.99
		√	67.70	96.71	88.00	48.14	9.81
